# Plasma proteomics of green turtles (*Chelonia mydas*) reveals pathway shifts and potential biomarker candidates associated with health and disease

**DOI:** 10.1093/conphys/coab018

**Published:** 2021-04-28

**Authors:** David P Marancik, Justin R Perrault, Lisa M Komoroske, Jamie A Stoll, Kristina N Kelley, Charles A Manire

**Affiliations:** 1 Department of Pathobiology, School of Veterinary Medicine, St. George’s University, True Blue, Grenada, West Indies; 2 Loggerhead Marinelife Center, 14200 US Highway One, Juno Beach, FL 33408, USA; 3 College of Natural Resources, University of Massachusetts Amherst, 230 Stockbridge Road, Amherst, MA 01003, USA

## Abstract

Evaluating sea turtle health can be challenging due to an incomplete understanding of pathophysiologic responses in these species. Proteome characterization of clinical plasma samples can provide insights into disease progression and prospective biomarker targets. A TMT-10-plex-LC–MS/MS platform was used to characterize the plasma proteome of five, juvenile, green turtles (*Chelonia mydas*) and compare qualitative and quantitative protein changes during moribund and recovered states. The 10 plasma samples yielded a total of 670 unique proteins. Using ≥1.2-fold change in protein abundance as a benchmark for physiologic upregulation or downregulation, 233 (34.8%) were differentially regulated in at least one turtle between moribund and recovered states. Forty-six proteins (6.9%) were differentially regulated in all five turtles with two proteins (0.3%) demonstrating a statistically significant change. A principle component analysis showed protein abundance loosely clustered between moribund samples or recovered samples and for turtles that presented with trauma (*n* = 3) or as intestinal floaters (*n* = 2). Gene Ontology terms demonstrated that moribund samples were represented by a higher number of proteins associated with blood coagulation, adaptive immune responses and acute phase response, while recovered turtle samples included a relatively higher number of proteins associated with metabolic processes and response to nutrients. Abundance levels of 48 proteins (7.2%) in moribund samples significantly correlated with total protein, albumin and/or globulin levels quantified by biochemical analysis. Differentially regulated proteins identified with immunologic and physiologic functions are discussed for their possible role in the green turtle pathophysiologic response and for their potential use as diagnostic biomarkers. These findings enhance our ability to interpret sea turtle health and further progress conservation, research and rehabilitation programs for these ecologically important species.

## Introduction

The ability to assess and monitor sea turtle health is an important component of many conservation programs. This includes performing health surveys to characterize population fitness, defining associations between turtle health and environmental conditions and examining the health of debilitated turtles undergoing veterinary care, rehabilitation and release (Herbst and Jacobson, 2003; Stringer *et al.*, 2010; Camacho *et al.*, 2013). These data can become important when developing policy changes, educational outreach and veterinary care protocols (Kock *et al.*, 1996; Deem *et al.*, 2001). Although sea turtle population numbers have declined worldwide throughout the past century (IUCN, 2020), recent increases in a number of select populations provide confidence that these continued conservation efforts can result in long-term successes (Mazaris *et al.*, 2017).

To date, clinical ante-mortem evaluation of sea turtle health largely involves haematology and blood biochemistry (Bolten and Bjorndal, 1992; Herbst and Jacobson, 2003; Deem *et al.*, 2009; Anderson *et al.*, 2011; Stacey and Innis, 2017). These assays are useful in many respects and continue to increase our understanding of disease in turtles. However, they are also complicated by the use of analytes and technologies that were originally developed for more traditional veterinary species. Analyte levels do not always represent the same physiologic change in sea turtles that they do in mammals and reference intervals vary between sea turtle species, geographical location, age, gender, breeding status and diet (Bolten and Bjorndal, 1992; Herbst and Jacobson, 2003; Deem *et al.*, 2009; Anderson *et al.*, 2011; Stacy *et al.*, 2018). This limitation can complicate data interpretation, especially with animals that may exhibit non-specific or subclinical signs of disease. Conservation, rehabilitation and research programs would benefit from a more complete understanding of the pathophysiology of disease in sea turtles and how to best differentiate healthy and diseased states diagnostically.

The pathophysiologic response of warm- and cold-blooded vertebrates is driven by coordinated upregulation and downregulation of plasma-derived inflammatory, metabolic and structural proteins (Marancik *et al.*, 2013; van Altena *et al.*, 2016; Geyer *et al.*, 2016). Monitoring how these proteins change in abundance and type over the course of disease can provide useful information regarding disease pathophysiology and development of clinical signs (Pierson *et al.*, 2018; Coleman *et al.*, 2020). Additionally, identifying individual proteins or panels of proteins that exhibit specific and reliable changes in abundance between healthy and diseased states can be useful as diagnostic biomarkers in humans (Geyer *et al.*, 2017). Similar conditions may apply to sea turtles and elucidation of these processes can improve our understanding of the sea turtle disease response and conditions associated with morbidity and mortality.

Along with publication of a reference genome (Wang *et al.*, 2013), the worldwide distribution (Jensen *et al.*, 2019) and relatively common inclusion of green turtles (*Chelonia mydas*) in research and rehabilitation settings make them a relevant model for studying disease pathophysiology in sea turtles. There are common clinical presentations of green turtles including trauma (including boat strikes, entanglement and shark bites), impaction with marine debris, fibropapillomatosis, buoyancy issues, cold stunning, parasitism and bacterial septicemia (Lackovich *et al.*, 1999; Torrent *et al.*, 2005; Stamper *et al.*, 2009; Flint *et al.*, 2015; Chapman *et al.*, 2017; Weisbrod *et al.*, 2020) that allow relatively wide application of pathophysiologic metrics compared with other turtle species. Additionally, these animals represent an important evolutionary bridge between cold- and warm-blooded animals (Work *et al.*, 2015). Elucidation of their pathophysiologic responses may yield valuable insights regarding immune system phylogeny in vertebrates.

The goal of this study was to characterize the plasma proteome of green turtles and to measure the differences in plasma protein type and abundance as turtles shifted from moribund to recovered states. A tandem mass tag (TMT) and shotgun-based nano liquid chromatography–tandem mass spectrometry (LC–MS/MS)-based platform was chosen for proteomic analysis. Isobaric tagging is often used to quantify proteins in clinical samples as it allows multiplexing of up to 10 different samples in one experiment, which significantly reduces technical variation (Li *et al.*, 2012). In this study, a global analysis of the green turtle proteome is presented and proteins with putative immunologic and physiologic functions are discussed for their role in health and disease of green turtles and potential exploration as diagnostic biomarkers.

## Materials and methods

### Clinical history

Five juvenile green turtles found debilitated off the eastern coast of Florida presented to the Loggerhead Marinelife Center (Juno Beach, FL) for treatment and rehabilitation (Table 1). Turtles were maintained outdoors in 1200–3600-L fibreglass tanks on a flow-through system utilizing natural sea water at ambient ocean temperature except that in winter the incoming water was heated when needed to maintain the water temperature >22°C. In addition to a daily diet of shrimp or squid and capelin (Table 1), turtles were supplemented with Mazuri® Sea Turtle Supplements and calcium (Risacal-D, Rising Pharmaceuticals Inc., Allendale, NJ).

**Table 1:** Clinical history, husbandry and plasma biochemistry data for five green turtles (*Chelonia mydas*) sampled at Loggerhead Marinelife Center

Turtle #	Diagnosis	Stranding date	Stranding mass (kg), SCL (cm)	Presenting biochemistry results (g/dL)	Release date	Release mass (kg), SCL (cm)	Diet
Turtle 1	Trauma	1/16/2018	2.1, 25.9	TP: 2.4 Albumin: 0.9 Globulins: 1.5	5/8/2018	2.7, 27.5	30% shrimp 70% capelin
Turtle 2	Intestinal floater	11/18/2017	23.4, 57.2	TP: 5.4 Albumin:1.5 Globulins: 3.9	1/23/2018	24.4, 57.8	33% shrimp 67% capelin
Turtle 3	Intestinal floater	1/7/2015	19.6, 50.1	TP: 5.4 Albumin: 1.5 Globulins: 3.9	12/2/2015	19.4, 50.4	33% shrimp 67% capelin
Turtle 4	Trauma	9/16/2019	2.8, 28.7	TP: 3.2 Albumin: 1.2 Globulins: 2.0	12/4/2019	2.9, 29.3	33% squid 67% capelin
Turtle 5	Shark trauma	10/6/2019	2.9, 29.1	TP: 2.3 Albumin: 1.0 Globulins: 1.3	12/11/2019	3.6, 31.4	30% squid 70% capelin

TP indicates total protein and SCLstandard indicates standard straight carapace length.

Blood samples were collected from the dorsal cervical sinus from each turtle upon admission and just prior to release (Table 1) using a 22 gauge, a 1-inch needle and a 3-cc syringe. The blood was immediately placed into 2-mL lithium heparin tubes and samples were centrifuged at ~4200 × g (5000 rpm) for 8 min using an LW Scientific C5 centrifuge. A 0.5-mL portion of plasma was stored at −80°C for up to 48 months to ensure stability (Townsend *et al.*, 2020) for proteomic analysis. A portion of the plasma sample collected at admission was used to run plasma biochemistry on an IDEXX Catalyst DX Chemistry Analyzer (Turtles 1, 2, 4, 5) or Abaxis VetScan VS2 (Turtle 3) biochemistry analyser (Table 1). For this study, total protein, albumin and globulin levels were analysed and compared with proteomic data collected from the same plasma sample.

### Proteomic analysis

A plasma proteomic analysis was performed at Cornell University Proteomics and Metabolomics Facility (Ithaca, NY). Plasma protein concentrations were estimated by the Bradford method (Bradford, 1976) and qualitatively visualized through standard 1D gel separation techniques.

Protein expression changes were quantified by TMT 10-plex profiling. In total, 100-μL aliquots of each of the 10 samples were labelled with TMT 10-plex tags. The mix tags labelled samples were constructed by first dimensional high pH RP separation of tryptic peptide mixtures by Ultimate3000 MDLC platform with built-in fraction collection option, autosampler and UV detection (Dionex, Sunnyvale, CA). The TMT tryptic peptides were reconstituted in 20-mM ammonium formate (NH_4_FA) pH 9.5 in water (buffer A) and loaded onto an XTerra® MS C18 column (3.5 μm, 2.1 × 150 mm, in water) (Waters Corp, Milford, MA) with buffer A and 80% acetonitrile (ACN)/20% 20-mM NH_4_FA (buffer B).

Liquid chromatography was performed using a gradient from 10% to 45% of buffer B for 30 min at a flow rate 200 μL/min. Fractions were collected at 1 min intervals in a 96-well plate and pooled based on UV absorbance at 214 nm. Fractions were pooled by disparate first dimension fractions (retention time multiplexing) using concatenation strategy. All pooled peptide fractions were dried and reconstituted in 2% ACN/0.5% formic acid for nano LC–MS/MS analysis.

Nano LC–MS/MS analysis was carried out on equal mixtures of tag labelled digests using an LTQ-Orbitrap Velos mass spectrometer (Thermo Fisher Scientific, San Jose, CA) equipped with nano ion source via high energy collision dissociation (HCD) and interfaced with an UltiMate3000 RSLC nano system (Dionex, Sunnyvale, CA). In total, 10-μL aliquots of each pH RP peptide fraction were injected onto a PepMap C18 trap column (5 μm, 300 μm × 5 mm) for desalting at 20 μL/min flow rate. Fractions were then separated on a PepMap C-18 RP nano column (3 μm, 75 μm × 15 cm) and eluted for 90 min in a gradient of 5%e 38% ACN in 0.1% formic acid at 300 μL/min followed by a 3-min ramping to 95% ACN-0.1% FA and a 5-min holding at 95% ACN-0.1% FA. The column was re-equilibrated with 2% ACN-0.1% FA for 20 min prior to the next run.

The eluted peptides were detected by Orbitrap through nano ion source containing a 10-mm analyte emitter (New Objective, Woburn, MA). The Orbitrap Velos was operated in positive ion mode with nano spray voltage set at 1.5 kV and source temperature at 275°C with nitrogen as the collision gas. Calibration was performed internally using the background ion signal at m/z 445.120025 as a lock mass or externally using a Fourier transform (FT) mass analyser. The instrument was run on data-dependent acquisition mode using FT mass analyser for survey MS scans of precursor ions followed by 10 data-dependent HCD-MS/MS scans for precursor peptides with multiple charged ions above a threshold ion count of 7500 with normalized collision energy of 45%. MS survey scans were conducted at a resolution of 30 000 FWHM at m/z 400 for the mass range of m/z 400e1400 and MS/MS scans were conducted at 7500 resolution for the mass range of m/z 100e2000. All data were acquired under Xcalibur 2.1 operation software (Thermo Fisher Scientific, San Jose, CA). All MS and MS/MS raw spectra data from TMT experiments were searched using PD2.3 (Thermo Fisher Scientific, Bremen, Germany) with SEQUEST HT searching engine. Processing workflow for reporter ions quantification in PD 2.3 was used for protein identification and protein relative quantitation analysis. Database searches were performed against the green turtle draft genome (https://www.genome.jp). Resulting proteins were considered confidently identified if more than two peptides were recognized, the protein false discovery rate was <0.01 and the protein was quantified in all samples evaluated.

### Bioinformatics and functional annotations

Gene Ontology (GO) terms for ‘biological processes’ associated with the identified proteins were examined further as these provide the most relevant information regarding disease pathogenesis. The longest amino acid sequence for each differentially regulated protein was extracted using a custom script and the DIAMOND protein aligner (Buchfink *et al.*, 2015) was used to identify pairwise alignments between our sequences and those of the curated UniProtKB/Swiss-Prot database (The UniProt Consortium 2019, accessed May 2020). DIAMOND blastp was run in more-sensitive mode and the single best alignment for each amino acid sequence was retained. UniProt identifiers generated from pairwise alignments were then used to retrieve GO biological process terms associated with each protein. Within the list of differentially regulated proteins (see statistical analysis details below), the number of proteins in moribund and healthy group associated with each GO term was quantified in R (R Core Team, 2017, v4.0.0) using the ggplot2 and tidyverse R packages (Wickham, 2016; Wickham *et al.*, 2019).

### Statistical analysis

A 1.2-fold change in protein abundance between moribund and recovered samples was used as a benchmark to indicate a physiologically significant change in protein abundance (Lee *et al.*, 2010; Xiaping *et al.*, 2012). GraphPad Prism 8.3 (San Diego, CA) and Qlucore Omics Explorer 3.5 (Lund, Sweden) was used to produce a heatmap and principle component analysis (PCA), respectively. Proteins that demonstrated a normal distribution and had ratios that were significantly different than 1 at a 95% CI were considered differentially expressed between moribund and recovered groups. Each protein was analysed individually without assuming a consistent standard deviation (SD). This analysis was limited to 44 proteins found to have consistent, 1.2-fold or greater change in all five turtles and was examined using GraphPad 8.3. A Spearman correlation was used to examine the relationships between protein abundance levels and plasma biochemistry values for total protein, albumin and globulin using IBM SPSS Statistics 26 software (SPSS, Inc., Chicago, IL). Differences were considered statistically significant when *P* < 0.05.

## Results

**Figure 1:**
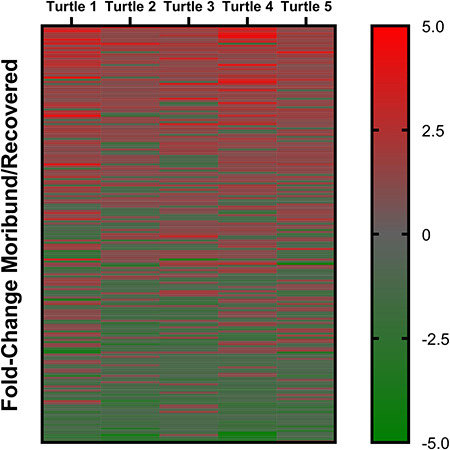
Heat map demonstrating 197 proteins differentially regulated in at least one rehabilitating green turtle (*Chelonia mydas*) with a 1.2–5.0-fold change between moribund and healthy states; each turtle is represented by a single column

**Figure 2:**
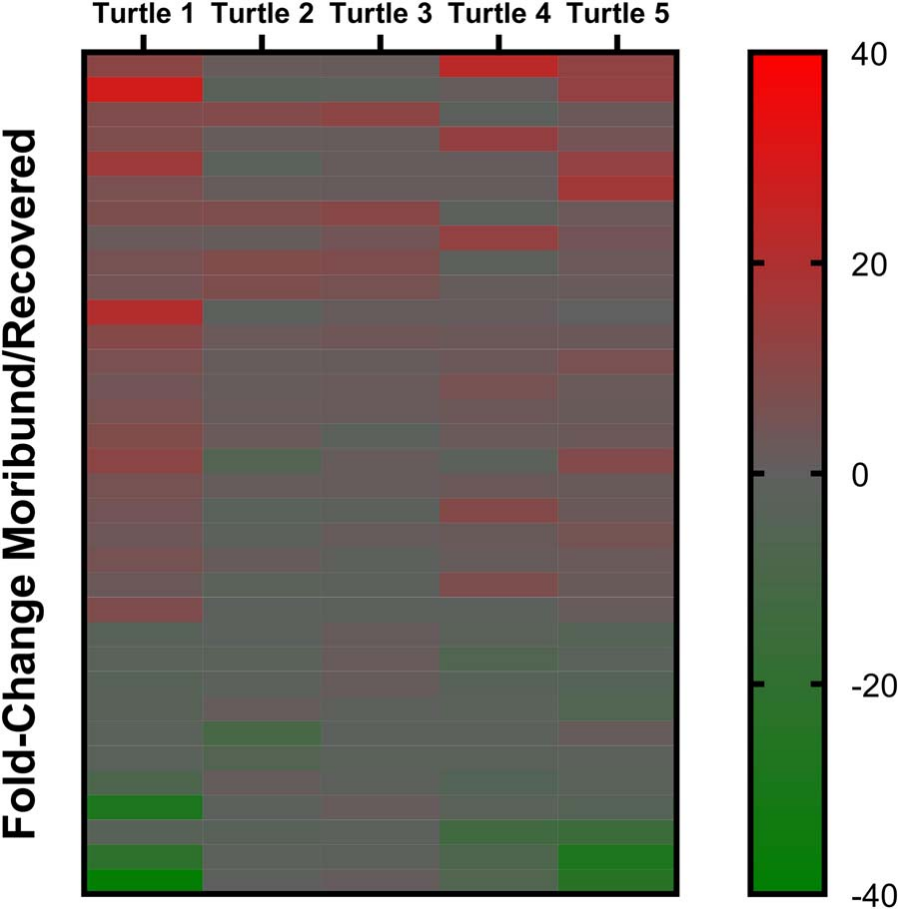
Heat map demonstrating 34 proteins differentially regulated in at least one rehabilitating green turtle (*C. mydas*) with ≥5.0-fold change between moribund and healthy states

**Figure 3:**
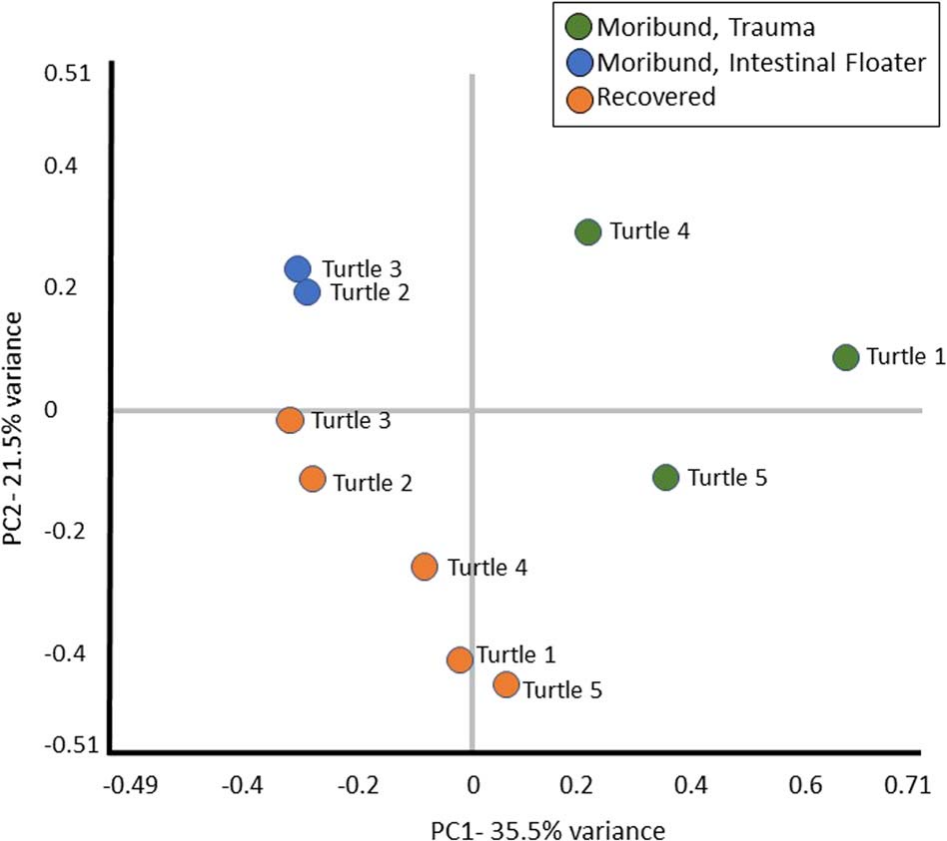
PCA comparing proteomic profiles between individual rehabilitating green turtles (*C. mydas*) and conditions based on 233 differentially regulated proteins

The 10 plasma samples yielded a total of 488 proteins identified from 8102 unique peptides (Supplemental File 1). In total, 231/488 (47.3%) proteins were interpreted as having undergone a physiologically significantly change in abundance in at least one turtle between moribund and recovered states. This included 197 proteins that exhibited a ±1.2–5.0-fold change (Fig. 1) and 34 proteins with a ±5.1-fold change or higher (Fig. 2). A PCA showed protein abundance levels loosely clustered for turtles that were either moribund or recovered and based on whether turtles presented with trauma or as intestinal floaters (Fig. 3).

**Figure 4:**
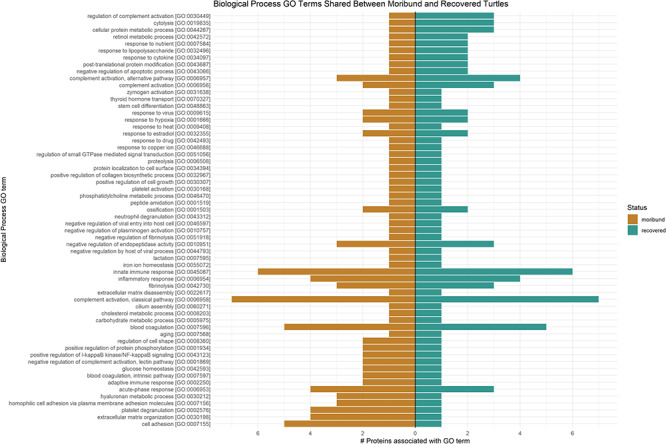
Comparison of proteins in samples from moribund and recovered rehabilitating green turtles (*C. mydas*) associated with a given biological process GO term

GO terms associated with biologic processes, molecular function and/or cellular component were obtainable for 169/231 (73.2%) proteins (Supplemental File 2). The GO terms associated with biologic processes were further examined for their applicability for defining pathophysiologic mechanisms occurring in moribund turtle samples. A total of 87 proteins matched to biologic process GO Terms (Supplemental File 2). Sixty of these proteins were shared between moribund and recovered samples (Fig. 4). Moribund turtle samples had a relatively higher number of proteins that matched with GO terms associated with various inflammatory processes such as GO:0043123 Positive Regulation of I-Kappa B kinase/NF-Kappa B signalling, GO:0007597 Blood coagulation, intrinsic pathway, GO:0002250 Adaptive Immune Responses, GO:0006953 Acute-phase Response and GO:002576 Platelet Degranulation. In recovered turtle samples, this included a relatively higher number of proteins associated with select metabolic processes such as GO:0044267 Cellular Protein Metabolic Processes, GO:0007584 Response to Nutrient, GO:0043066 Negative Regulation of Apoptotic Processes and GO:0042572 Retinol Metabolic Processes (Fig. 4).

In total, 44/488 (9.0%) proteins demonstrated consistent upregulation or downregulation patterns in all five moribund turtle samples with at least one sample exhibiting a physiologically significant change in abundance (Table 2). Thrombospondin-1 isoform X1 and vitamin K-dependent protein Z additionally demonstrated a statistically significant difference in abundance between moribund and recovered states (adjusted *P* = 0.04) (Table 2).

**Table 2:** Protein abundance mean and SD for green turtles (*Chelonia mydas*) demonstrating equivalent expression trends in all five turtles and at least 1.2-fold difference in abundance from moribund to healthy states in at least one turtle

Accession number	Protein	Abundance moribund mean ± SD	Abundance recovered mean ± SD	Fold change mean ± SD	Abundance difference *P*-value
XP_007067898.1	Protein S100-A12	328.4 ± 326.0	37.1 ± 13.3	9.86 ± 8.69	
XP_007063716.1	Complement factor H-related protein 2	117.9 ± 100.4	24.7 ± 6.0	5.10 ± 4.56	
XP_007053239.1	14-3-3 protein theta	40.3 ± 23.6	10.4 ± 3.6	4.20 ± 2.89	
XP_027684798.1	Betaine–homocysteine S-methyltransferase 1	743.7 ± 889.3	129.6 ± 90.1	5.27 ± 6.50	
XP_007069697.1	Triosephosphate isomerase	115.0 ± 122.6	26.5 ± 10.3	3.68 ± 2.56	
XP_007070993.1	Actin, cytoplasmic 2	50.6 ± 27.4	16.9 ± 3.4	3.04 ± 1.74	
XP_007066059.1	Glycogen phosphorylase, liver form	65.1 ± 43.7	26.3 ± 3.8	2.59 ± 1.88	
XP_007071883.1	Isocitrate dehydrogenase [NADP]	133.7 ± 75.2	57.7 ± 8.2	2.72 ± 1.36	
XP_027679113.1	Transaldolase	162.4 ± 64.8	84.2 ± 16.3	2.26 ± 1.01	
XP_027683601.1	Fibrinogen-like protein 1-like protein	936.3 ± 297.6	589.2 ± 219.0	2.25 ± 0.99	
XP_007067024.1	Heat shock cognate 71 kDa protein	468.1 ± 227.2	205.8 ± 26.3	2.14 ± 1.09	
XP_007065523.1	Fibrinogen-like protein 1-like protein	285.5 ± 96.5	171.6 ± 73.4	2.22 ± 1.21	
XP_007061563.1	Elongation factor 1-alpha 1	0.9 ± 0.3	0.5 ± 0.1	2.26 ± 1.10	
XP_027677046.1	Plastin-2	378.7 ± 200.2	198.6 ± 62.6	2.02 ± 1.04	
XP_027684709.1	Collagen alpha-1(XII) chain	234.4 ± 119.2	102.5 ± 34.5	1.70 ± 0.68	
XP_027690646.1	Cytosolic non-specific dipeptidase	138.8 ± 42.5	89.9 ± 20.4	1.77 ± 0.63	
XP_027682462.1	Coagulation factor XIII A chain	172.2 ± 45.1	101.8 ± 27.2	1.65 ± 0.59	
XP_007070269.1	Alpha-enolase	58.3 ± 35.8	27.1 ± 13.3	1.93 ± 0.80	
XP_007057340.2	Alpha-1-acid glycoprotein 2-like	166.3 ± 81.7	77.3 ± 23.9	1.55 ± 0.38	
XP_027687337.1	Thrombospondin-1 isoform X1	104.6 ± 23.4	44.9 ± 17.4	1.72 ± 0.31	0.04
XP_007065783.1	Complement component C6	108.3 ± 23.0	84.2 ± 13.6	1.29 ± 0.26	
XP_027678437.1	Lysosome-associated membrane glycoprotein 2 isoform X1	86.1 ± 35.9	59.7 ± 16.1	1.42 ± 0.26	
XP_027675100.1	von Willebrand factor	335.0 ± 90.2	220.1 ± 85.4	1.69 ± 0.81	
XP_007071418.2	Carboxypeptidase N subunit 2 isoform X1	130.0 ± 22.6	103.4 ± 23.9	1.28 ± 0.18	
XP_027690298.1	Complement C1r subcomponent	165.8 ± 51.8	107.4 ± 17.9	1.55 ± 0.48	
XP_027684438.1	Immunoglobulin lambda variable 5-39	355.0 ± 231.2	223.0 ± 130.1	1.54 ± 0.18	
XP_027679862.1	Target of Nesh-SH3 isoform X1	85.8 ± 30.3	61.3 ± 16.1	1.38 ± 0.17	
XP_027687083.1	Integrin beta-2	181.6 ± 60.3	101.9 ± 10.1	1.75 ± 0.43	
XP_007054804.1	Membrane primary amine oxidase	192.4 ± 35.8	157.3 ± 27.8	1.23 ± 0.19	
XP_007072391.1	Complement C1s subcomponent	119.5 ± 34.0	79.7 ± 14.6	1.53 ± 0.50	
XP_027690386.1	Integrin alpha-L	110.8 ± 53.4	60.9 ± 7.3	1.78 ± 0.75	
XP_007069327.1	Phosphatidylcholine-sterol acyltransferase	208.4 ± 96.0	304.4 ± 94.1	−1.63 ± 0.66	
XP_007071006.1	Apolipoprotein E	59.6 ± 34.7	94.8 ± 44.3	−1.27 ± 0.21	
XP_007065578.1	Serum albumin	87.0 ± 12.1	108.6 ± 8.4	−1.32 ± 0.27	
XP_007053770.1	Mannan-binding lectin serine protease 2	110.1 ± 23.8	151.8 ± 23.8	−1.34 ± 0.35	
XP_027685684.1	Protein AMBP	88.8 ± 37.6	111.2 ± 30.4	−1.40 ± 0.17	
XP_007057844.1	Vitronectin	83.6 ± 26.2	136.3 ± 24.5	−1.60 ± 0.31	
XP_027673810.1	Alpha-2-macroglobulin	108.3 ± 32.8	138.4 ± 30.9	−1.73 ± 0.71	
XP_007060524.1	Apolipoprotein A-I	90.9 ± 66.9	155.9 ± 69.6	−1.76 ± 0.64	
XP_007057023.2	Ovotransferrin	49.0 ± 10.5	77.9 ± 20.4	−2.13 ± 1.44	
XP_007063956.1	Vitamin K-dependent protein Z	39.1 ± 9.5	88.9 ± 20.3	−2.31 ± 0.47	0.04
XP_027688886.1	Apolipoprotein A-IV-like	56.2 ± 26.2	112.1 ± 55.7	−2.22 ± 1.07	
XP_007060525.1	Apolipoprotein A-IV	60.4 ± 41.1	287.8 ± 171.4	−7.22 ± 6.34	
XP_007061965.1	Collagen alpha-1(I) chain isoform X1	8.0 ± 1.5	93.1 ± 98.9	−11.94 ± 12.22	

Proteins with no *P*-value listed were not statistically significantly different between moribund and healthy states.

Abundance levels of 48/488 (9.8%) proteins in moribund turtles significantly correlated with total protein (18), albumin (32) and/or globulin (18) levels (Table 1) quantified by biochemical analysis. Statistical confidence in individual correlation coefficients was low due to the small sample size (Supplemental File 3) and thus general correlation patterns were examined. The majority of proteins, 37/48 (77.1%), negatively correlated with biochemical analyte levels. Proteins that correlated with total protein also tended to correlate with globulins (16/48, 33.3%).

## Discussion

There is a growing need to understand and more accurately interpret sea turtle health for rehabilitation, research and conservation goals. The objective of this study was to characterize the plasma proteome of green sea turtles and to describe changes in protein type and abundance between moribund and recovered states. Over 450 proteins were identified in green sea turtle plasma and ~9% of these proteins were consistently upregulated or downregulated in all turtles as they recovered in rehabilitation. This includes proteins with putative roles in the acute-phase response, innate and adaptive immunity and metabolism. Elucidating these patterns contributes to our understanding of the pathophysiologic response in sea turtles and provides another foundation for exploring biomarkers of health and disease.

Heat maps showed roughly one-half of identified proteins demonstrated some level of physiologic change between moribund and recovered states but trends were not consistent between all turtles. A coordinated response was more apparent in proteins that exhibited a 1.2-fold to 5.0-fold change in abundance and trends became less consistent when proteins reached >5.0-fold change. The differences in responses between turtles was not unexpected as individual biologic variability has been well described as a limitation in clinical proteomic studies in humans (Yeh *et al.*, 2017). We aimed to reduce this variable by using paired moribund and healthy samples from this same individual for this relatively small sample size; however, the pathophysiologic response of each turtle was likely influenced by unique clinical timelines and disease characteristics. Additionally, environmental variables play a significant role in poikilothermic sea turtles as physiologic condition can be affected by water temperature, reproductive status, turtle size and diet (Bolten and Bjorndal, 1992; Bullock, 1995; Deem *et al.*, 2009, Stacy *et al.*, 2018). Although there were no overt patterns appreciated when proteomic results were examined in relation to carapace length and the seasonal variance, the sample size was too small to provide a statistically relevant comparison; however, these factors should be considered in future studies. Despite the small sample size and unique clinical characteristics, trends were still evident between turtles and PCA showed clustering not only of moribund and recovered turtles but also between turtles that were diagnosed with trauma or with gas accumulation in the intestine. This provides evidence for conserved and likely aetiology-specific pathophysiologic responses in debilitated turtles.

GO terms categorized a number of differentially regulated proteins with purported immunologic and metabolic roles. As anticipated, the number of proteins corresponding with inflammatory-associated GO terms was higher in moribund turtle samples compared with recovered turtle samples. This included annotations for the acute-phase (GO:0006953) and adaptive immune responses (GO:0002250, GO:0043123, GO:0001934). Unexpectedly, recovered turtle samples demonstrated a higher number of GO terms associated with complement activity (GO:0030449, GO:0006957, GO:0006956) and, correspondingly, moribund turtles showed a higher number of proteins associated with negative regulation of complement activation (GO:0001869). Individually, complement components C6 and C1s, complement C1r subcomponent and regulatory complement factor H-related protein were upregulated in all five moribund turtle samples, indicating that there was an increase in at least some complement factors. The complement system plays a universal role in defence against pathogens and clearance of apoptotic and injured cells in vertebrates (Sunyer and Lambris, 1998; Noris and Remuzzi, 2013) and upregulation in moribund turtles would be expected. As a whole, the complement system has not been well characterized in sea turtles and there is evidence that species-specific differences may exist in structure in turtles (Baker *et al.*, 2019). The relatively high number of GO terms and proteins associated with complement activity, including differentially regulated complement components, indicates that this system plays an important role in the immunologic response of sea turtles and warrants further characterization.

There were loose groupings within the PCA between the three turtles that presented with trauma as well as between the two turtles that presented with gas accumulation in the intestine. Within this analysis, a number of proteins stood out as being more greatly associated with one group versus the other, although the small sample size precluded statistical analysis. For example, reporter ion S100-A12 abundance was similar in all recovered turtles (37.1 ± 13.3) regardless of disease aetiology but demonstrated a 1.9-fold increase in moribund samples from intestinal floaters and a 15.2-fold increase in turtles presenting with trauma. S100-A12 is a calcium binding, pro-inflammatory protein (Khorramdelazad *et al.*, 2015). Levels serve as a biomarker for a number of inflammatory conditions (Meijer *et al.*, 2012) and are significantly associated with cellular damage, trauma and sepsis in humans (Meijer *et al.*, 2012; Dubois *et al.*, 2019). The higher plasma levels observed in turtles with traumatic injury are consistent with the association of S100-A12 with trauma and possibly secondary bacterial infection of the wounds. Similarly, complement component C6, complement C1r subcomponent and collagen alpha-1(XII) chain abundance were 1.5-fold, 1.6-fold and 2.5-fold higher in turtles with trauma compared with intestinal floaters. Whether this is associated with the level of injury, possible secondary bacterial infections (Hecke *et al.*, 1997) or other factors requires further study.

There were a number of proteins upregulated in moribund turtle samples with putative roles in limiting the inflammatory response. Thrombospondin-1 isoform X1 was the most significantly changed protein (*P* = 0.04) with a 1.7-fold increase in abundance between recovered and moribund turtle samples. Thrombospondin-1 has well-defined immunomodulatory roles (Grimbert *et al.*, 2006) and is predictive in a wide variety of infectious and non-infectious syndromes in humans (Ohyama *et al.*, 2012; Suh *et al.*, 2012; Gao *et al.*, 2015; Decker *et al.*, 2020). Its level of statistical significance in this study suggests it may be substantially regulated between healthy and moribund states and warrants examination as a possible non-specific biomarker of disease. Other immunomodulatory proteins that were increased in all moribund turtle samples included the acute-phase protein alpha-1-acid glycoprotein (Hochepied *et al.*, 2003) and heat shock cognate 71 kDa protein, which has been shown to respond to protect cells from physiological stressors (Giuliano *et al.*, 2011). Previous studies have shown upregulation of heat shock proteins in experimentally heat-stressed loggerhead turtle embryos suggesting their role as biomarkers for thermotolerance (Tedeschi *et al.*, 2016). The results described herein suggest heat shock proteins may also be increased in turtles undergoing other pathophysiologic stressors or injury.

A large number of proteins identified in the green turtle proteome had putative roles associated with hemostasis. Moribund samples had a higher number of proteins associated with the intrinsic coagulation cascade (GO:0007597) and platelet degranulation (GO:0002576), although protein numbers that matched to the coagulation cascade (GO:0007596) were similar between both groups. The anti-coagulant vitamin-K dependent protein Z was statistically higher in recovered turtles while two fibrinogen-like proteins and von Willebrand factor were upregulated in moribund samples. Upregulation of hemostatic factors during disease highlights the shared signal pathways between coagulation and inflammation (Margetic, 2012). Blood coagulation is one of the oldest evolutionarily conserved processes in animals, occurring before the appearance of both teleosts and tetrapods over 430 million years ago (Davidson *et al.*, 2003). However, there are differences between sea turtle coagulation and that described in other animals. The intrinsic/contact activation pathway of the coagulation cascade appears to be non-functional in sea turtles due to lack of factors XI and XII (Soslau *et al.*, 2004). Upregulation of coagulation factor XII alpha chain in moribund samples, which represents an intermediary step in the production of activated factor XIIa (MacQuarrie *et al.*, 2011), may not be representative of factor XII itself or the peptide sequence was possibly annotated incorrectly. The impact that hemostatic changes have on pathophysiologic responses and morbidity in green turtles may be substantial as indicated by the breadth of changes observed in the plasma proteome.

There was an overrepresentation of carrier proteins upregulated in recovered turtle samples, including apolipoprotein E, apolipoprotein A-I, apolipoprotein A-IV-like, apolipoprotein A-IV and albumin. Albumin levels and to a lesser extent apolipoproteins, trended lower in turtles presenting with trauma than those categorized as intestinal floaters that may correspond with the severity of tissue injury or length of anorexia. High-density lipoproteins have roles in transport, metabolism and tissue repair (Weisgraber, 1994; Kaneko, 1997) and have been designated negative acute-phase proteins in numerous warm-blooded species (Lindhorst *et al.*, 1997; Burger and Dayer, 2002; Carpintero *et al.*, 2005). Assuming that plasma levels in recovered turtles are representative of homeostatic levels, decreased carrier protein abundance during moribund states likely represents a shift away from metabolic and anabolic processes (Sung *et al.*, 2004). Further characterization may provide guidance for nutritional and therapeutic support for moribund sea turtles.

In general, the agreement between proteomic and biochemical results was relatively low in moribund samples with only ~7% of the proteome correlating with total protein, albumin and/or globulin levels. This included 18/488 (3.7%) of proteins that correlated with total protein. This may suggest that in moribund states, total protein levels are only affected by a select number of high-abundance proteins. The majority of correlations were negative, with total protein and globulin showing similar correlation patterns. This is consistent with previous data in loggerhead turtles that demonstrates an underestimation of albumin using bromocresol green biochemistry methods (Müller and Brunnberg, 2010). A larger sample size is needed to confirm these trends. Samples from recovered states were not included in this analysis as matched proteomic and biochemical data were not collected from the same turtle.

There may be other pathophysiologically important annotations or proteins that were not highlighted in this study based on the statistical parameters utilized. Approximately 40% of proteins identified in the green turtle proteome did not significantly match to GO annotations. Confident assessment of biologic, cellular and structural roles within a global analysis requires a more comprehensive linkage with the green turtle genome. Additionally, individual proteins may have been excluded from the analysis due to inconsistent expression patterns. Serum amyloid A rose almost 100-fold in one moribund sample but fell 100-fold in another. The large SD and contradictory trends made it difficult to confidently conclude it as being pathophysiologically responsive although it is well known as a highly conserved acute-phase protein of vertebrates (Sack, 2018). Modifications to the methods used herein may also provide a more thorough assessment of the green turtle proteome. For example, abundant proteins were not depleted prior to TMT analysis. Protein depletion can uncover smaller abundant proteins that may be otherwise obscured by the major plasma proteins (Geyer *et al.*, 2016) but it is also associated with non-specific loss of protein (Geyer *et al.*, 2016), which is why it was not performed in this study. Optimally, analyses should include both methods for complete characterization of the plasma proteome.

The number of differentially regulated proteins identified in the green turtle plasma proteome that have described inflammatory, immunologic and physiologic roles is intriguing. This sets the stage to further explore mechanisms of disease and to identify potential biomarkers using more targeted and protein-specific methodologies for analytes of interest. Further studies utilizing a larger sample size and controls for variables such as disease aetiology, timeframe, turtle size and other environmental effects are needed to examine changes in the context. Additionally, comparisons between sea turtle species may provide additional biomarker data regarding conserved and unique disease responses. Further progress in this area will help drive our understanding of sea turtle pathophysiology may elucidate important biomarkers of disease.

## Supplementary Material

Supplemental_Files_coab018
